# Protein Phosphorylation in Serine Residues Correlates with Progression from Precancerous Lesions to Cervical Cancer in Mexican Patients

**DOI:** 10.1155/2020/5058928

**Published:** 2020-04-02

**Authors:** Juan Ramón Padilla-Mendoza, Arturo Contis-Montes de Oca, Mario Alberto Rodríguez, Mavil López-Casamichana, Jeni Bolaños, Laura Itzel Quintas-Granados, Octavio Daniel Reyes-Hernández, Fabiola Fragozo-Sandoval, Aldo Arturo Reséndiz-Albor, Claudia Vanessa Arellano-Gutiérrez, Israel López-Reyes

**Affiliations:** ^1^Departamento de Infectómica y Patogénesis Molecular, Centro de Investigación y de Estudios Avanzados (CINVESTAV-IPN), Ciudad de México 07360, Mexico; ^2^Universidad Autónoma de la Ciudad de México (UACM), Plantel Cuautepec, Ciudad de México 07160, Mexico; ^3^Sección de Estudios de Posgrado e Investigación, Escuela Superior de Medicina, Instituto Politécnico Nacional (ESM-IPN), Ciudad de México 11340, Mexico; ^4^Posgrado en Ciencias Genómicas, Universidad Autónoma de la Ciudad de México (UACM), Plantel del Valle, Ciudad de México 03100, Mexico; ^5^Unidad de Estudios Superiores de Tultitlan, Universidad Mexiquense del Bicentenario, Estado de México 54910, Mexico; ^6^Unidad Multidisciplinaria de Investigación Experimental (UMIEZ), Facultad de Estudios Superiores Zaragoza, Universidad Nacional Autónoma de México (UNAM), Ciudad de México 09230, Mexico; ^7^Clínica de Displasias y Anatomía Patológica, Hospital Juárez de México, Ciudad de México 07760, Mexico; ^8^Laboratorio de Bioquímica Farmacológica, Escuela Nacional de Ciencias Biológicas, Instituto Politécnico Nacional (ENCB-IPN), Ciudad de México 11340, Mexico

## Abstract

Protein phosphorylation is a posttranslational modification that is essential for normal cellular processes; however, abnormal phosphorylation is one of the prime causes for alteration of many structural, functional, and regulatory proteins in disease conditions. In cancer, changes in the states of protein phosphorylation in tyrosine residues have been more studied than phosphorylation in threonine or serine residues, which also undergo alterations with greater predominance. In general, serine phosphorylation leads to the formation of multimolecular signaling complexes that regulate diverse biological processes, but in pathological conditions such as tumorigenesis, anomalous phosphorylation may result in the deregulation of some signaling pathways. Cervical cancer (CC), the main neoplasm associated with human papillomavirus (HPV) infection, is the fourth most frequent cancer worldwide. Persistent infection of the cervix with high-risk human papillomaviruses produces precancerous lesions starting with low-grade squamous intraepithelial lesions (LSIL), progressing to high-grade squamous intraepithelial lesions (HSIL) until CC is generated. Here, we compared the proteomic profile of phosphorylated proteins in serine residues from healthy, LSIL, HSIL, and CC samples. Our data show an increase in the number of phosphorylated proteins in serine residues as the grade of injury rises. These results provide a support for future studies focused on phosphorylated proteins and their possible correlation with the progression of cervical lesions.

## 1. Introduction

Protein phosphorylation is a posttranslational modification (PTM) carried out on tyrosine, threonine, and serine residues [1, 2], which regulates protein functions and signaling pathways that orchestrate a variety of cellular processes, such as cell growth, differentiation, and apoptosis [3–6]. This PTM is essential for normal cellular processes, while abnormal phosphorylation is one of the prime causes for alteration of many structural, functional, and regulatory proteins in disease conditions [5, 7, 8]. In cancer, changes in the states of protein phosphorylation in tyrosine residues have been more studied than phosphorylation in the other two residues, although they also undergo changes with greater predominance [2]. Particularly, the protein phosphorylation in serine residues leads to the formation of multimolecular signaling complexes that regulate diverse biological processes, for example, DNA processing and repair, apoptosis, cell division, proliferation, and differentiation; nevertheless, in pathological conditions such as tumorigenesis, abnormal serine phosphorylation may result in the deregulation of signaling pathways responsible of proliferation and apoptosis inhibition [9–11].

The identification of serine phosphorylated proteins that participate in deregulate cellular functions in cancer would be of great help to know their functions within the cells [2]. The use of western blot assays with antibodies against serine phosphorylation may allow the detection of phosphorylated proteins [12, 13], which can be identified by mass spectrometry (MS). Afterwards, antibodies against these phosphorylated proteins could be used to evaluate the changes in concentration and in phosphorylation states of them during cancer development [14–16]. Thus, the detection of these changes could be useful for therapeutic, diagnostic, and/or prognostic applications [17, 18].

Cervical cancer (CC), the main neoplasm associated with human papillomavirus (HPV) infection [19], is the fourth most frequent cancer worldwide; in 2018, it affected 570,000 women and caused more than 311,000 deaths [20]. In Mexico, CC is the third most common cancer in women with 7689 new cases reported in 2018 [21]. Persistent infection of the cervix with high-risk human papillomaviruses (HPV-HR) produces precancerous lesions starting with low-grade squamous intraepithelial lesions (LSIL), progressing to high-grade squamous intraepithelial lesions (HSIL) until CC is generated [22, 23]. A previous study [24] showed the relevance of protein phosphorylation in tyrosine residues within the carcinogenesis progression of CC and suggested to perform new studies on the implication of phosphoproteins in threonine and serine residues on this cancer.

Here, we compared the proteomic profile of protein phosphorylation in serine residues from healthy (control), LSIL, HSIL, and CC samples obtained using cervical cytobrushes. Our data show an increase in the number of phosphorylated proteins in serine residues as the grade of injury rises. We focused in HSP27, Clusterin, KRT8, and KRT19 proteins, and we proposed that their phosphorylation in serine residues could be related in the progression to CC. Results provide a support for future studies centered on these proteins and their possible correlation with the progression of cervical lesions in patients infected with HPV-HR.

## 2. Material and Methods

### 2.1. Samples

Cervical cytobrushes and biopsies from women diagnosed pathologically with low-grade squamous intraepithelial lesions (LSIL), high-grade squamous intraepithelial lesions (HSIL), cervical cancer (CC), and healthy patients (Control) were provided from Hospital Juárez de México (Mexico City, México) and Clínica Integral de la Mujer (Mexico City, México). The present study followed the Declaration of Helsinki for the medical protocol and ethics, and the institutional committee of research and ethics approved the study (registration no. HMJ 2231/13-B). Expert colposcopists performed all examinations according to the terminology of the International Federation of Gynecology and Obstetrics (FIGO) [25]. Samples of a total of 50 patients with an age range between 23 and 56 years (13 healthy patients, 23 LSIL patients, 4 HSIL patients, and 10 CC patients) were evaluated.

### 2.2. Immunohistochemistry

Biopsy specimens from the cervix with LSIL, HSIL, CC, and healthy women were collected in a formalin-fixed buffer and stored at 4°C until further processing. Then, samples were embedded in paraffin, and tissue sections (5 *μ*m) were deparaffinized and rehydrated by passage through xylene and graded ethanol solutions. Slides were treated with 3% hydrogen peroxidase containing 0.03 sodium azide in TBS (Tris buffer solution) during 5 min followed by microwave antigen retrieval at 100°C for 10 min in DAKO target retrieval solution in a H2800 Microwave processor. Next, serial sections were incubated in 0.05% albumin in TBS for 30 min at room temperature to block nonspecific protein binding. Afterwards, monoclonal antiphosphoserine antibody (Sigma Aldrich, USA), or anti-P16 antibody (Santa Cruz Biotechnology, USA), were applied to sections at 1 : 50 dilution for 60 min at 4°C. Mouse IgG Ready-To-Use was used as a negative control. DAKO EnVision+System-HRP labelled polymer anti-mouse was used as the detection system and colorized by DAB (DAKO Corporation, USA). Samples were counterstained with Mayer's modified hematoxylin (Poly Scientific, USA) before mounting and viewed under an optical microscope (Olympus BX51). Images were recorded with a DP70 digital camera (Olympus Optical Co. Ltd., Japan).

### 2.3. Protein Extracts

Tissue samples gotten from cytobrushes were placed in isotonic saline solution, in the presence of protease and phosphatase inhibitors (Sigma Aldrich, USA). Then, they were sonicated at 100 W of tension, and proteins were precipitated with 10% trichloroacetic acid (TCA) at ratio 1 : 1 for 15 min on ice. Samples were centrifuged at 14,000 g for 5 min at 4°C and washed with 95% ethanol, followed by 75% ethanol; pellets were resuspended in extraction buffer (8 M urea, 2% CHAPS, 50 mM DTT, and 100 mM NaCl); and protein concentration was quantified by the Lowry method with bovine serum albumin (Sigma Aldrich, USA) as a reference standard [26].

### 2.4. Western Blot

Proteins were separated by 12% SDS-PAGE and transferred onto 0.22 *μ*m nitrocellulose membrane (Bio-Rad, USA) for 30 min at 0.4 Amp in a semidry transfer cell (Bio-Rad, USA). Afterwards, membranes were blocked with 3% of bovine serum albumin (Sigma Aldrich, USA) in TBS pH 7.4 at room temperature for 2 h. Then, membranes were incubated overnight at 4°C with the monoclonal antiphosphoserine antibody (1 : 1000, Sigma Aldrich, USA) and, after washing four times with TBS (10 min each), for another 4 h with a goat anti-IgG mouse conjugated to HRP (1 : 1000; Thermo Scientific, USA) at room temperature. Membranes were washed four times with TBS 1x (10 min each) and revealed with ECL Plus (GE Healthcare, USA). Images were acquired using a UVP transilluminator system **(**MyECL Imager, Thermo Scientific, USA). As a loading control, same membranes were incubated with a mouse monoclonal antibody against GAPDH (1 : 3000, Abcam, USA). Then, for semiquantitative analysis, the GAPDH and some phosphoproteins bands that showed an apparent increase during tumorigenesis progression were evaluated by densitometry using the ImageJ software (NIH, USA). All experiments were performed by duplicate. The relative expression in healthy tissues of these phosphoproteins was arbitrary taken as the unit. The differences of relative expression in cervical lesions were determined by Student's *t*-test, considering *p* values < 0.05 as statistically significant.

### 2.5. Two-Dimensional Electrophoresis (2DE)

For 2DE, 1 mg of protein extract each group was cleaned using the 2D-Clean Up Kit (GE Healthcare, USA) following the manufacturer's instructions. Protein pellets were resuspended with 125 *μ*L of Ready-Prep Rehydration buffer (Bio-Rad, USA) containing 8 M urea, 2% CHAPS, 50 mM DTT, 0.2% (*w*/*v*) Bio-Lyte 3-10 ampholytes, and bromophenol blue traces and supplemented with a protease and phosphatase inhibitor (Sigma Aldrich, USA). Then, samples were loaded onto 7 cm IPG strips (Bio-Rad, USA) pH 3-10 (linear gradient) and maintained for 16 h at 25°C for rehydration. After, isoelectric focusing was performed at 500 V (hold) for 1 h, 1000 V (lineal) for 0.5 h, 4000 V (gradient) for 1 h and 4,000 V (gradient) to complete 18,000 V/h, and 500 V (Hold), using the PROTEAN® i12™ IEF system (Bio-Rad, USA). Afterwards, strips were treated for 15 min at room temperature in equilibration buffer containing 75 mM Tris-HCl pH 8.8, 6 M urea, 30% glycerol (*v*/*v*), 2% SDS (*w*/*v*), 0.002% bromophenol blue, and 64 mM DTT. Then, strips were incubated for 15 min in the same solution containing 135 mM iodoacetamide. After reduction/alkylation procedure, IPG strips were loaded onto the 12% SDS-PAGE and electrophoresed at 100 V for 1.5 h. Gels were submitted to western blot as described above. Images were recorded using the Imager Scanner instrument (Amersham Biosciences, USA). Experiments were run by duplicate to obtain the technical and biological replicates.

### 2.6. Identification Phosphorylated Protein by Mass Spectrometry Analysis

Protein extract (30 *μ*g) for each condition (in biological triplicates) was subjected to 12% SDS-PAGE allowed to advance for about 1 cm within the gel; the resulting gel fragments were digested with trypsin according to Shevchenko et al. [27]. Afterwards, 1 *μ*L of 1 pmol·*μ*L^−1^ of alcohol dehydrogenase 1 (ADH1) of *Saccharomyces cerevisiae* (Uniprot accession: P00330) (Waters, USA) was added to all peptide samples to obtain a final concentration of 25 fmol·*μ*L^−1^ (internal standard). Then, 4 *μ*L of digested peptides (100 fmol of internal standard) for each biological sample was injected (in technical triplicates) into Symmetry C18 Trap V/M precolumn (Waters, USA):180 *μ*m × 20 mm, 100 Å pore size, 5 *μ*m particle size, desalted using as a mobile phase A, 0.1% formic acid (FA) in H_2_O and mobile phase B, and 0.1% FA in acetonitrile (ACN) under the followed isocratic gradient: 99.9% mobile phase A and 0.1% of mobile phase B at a flow of 5 *μ*L·min^−1^ during 3 min. Then, peptides were loaded and separated on a HSS T3 C18 column (Waters, USA): 75 *μ*m × 150 mm, 100 Å pore size, 1.8 *μ*m particle size; using an UPLC ACQUITY M-Class (Waters, USA) with the same mobile phases under the followed gradient: 0 min 7% B, 121.49 min 40% B, 123.15 to 126.46 min 85% B, and 129 to 130 min 7% B, at a flow of 400 nL·min^−1^ and 45°C. The spectra data were acquired in a mass spectrometer with electrospray ionization (ESI) and ion mobility separation (IMS) Synapt G2-S*i* (Waters, USA) operated in data-independent acquisition (DIA) using High-Definition-Multiplexed MS/MS (HDMS^E^) mode (Waters, USA). The tune page for the ionization source was set with the following parameters: 2.75 kV in the sampler capilar, 30 V in the sampling cone, 30 V in the source offset, 70°C for the source temperature, 0.5 Bar for the nanoflow gas, and 150 L·hr^−1^ for the purge gas flow. Two chromatograms were acquired (low- and high-energy chromatograms) in positive mode in a range of m/z 50-2000 with a scan time of 500 ms. No collision energy was applied to obtain the low-energy chromatogram, while for the high-energy chromatograms, the precursor ions were fragmented in the transfer using a collision energy ramp of 19-55 V. Synapt G2-S*i* was calibrated with [Glu1]-Fibrinopeptide fragments, through the precursor ion [M + 2H]^2+^ = 785.84261 fragmentation of 32 eV with a result less than 1.5 ppm across all MS/MS measurements.

### 2.7. Comparative Analysis of Phosphorylated Proteins in Serine Residues

Generated ∗.raw files containing MS and MS/MS spectra were deconvoluted, compared, identified [28], and quantified using Progenesis QI for Proteomics *v*4.1 software [29] (Waters, USA) against a reversed *Homo sapiens* (downloaded from Uniprot, 73099 protein sequences, last modification 27th June 2018) plus ADH (accession P00330) ∗.fasta database [30]. The parameters used for the protein identification were trypsin as an enzyme and one missed cleavage allowed; carbamidomethyl (C) as a fixed modification and oxidation (M), amidation (N-term), deamidation (N, Q), oxidation (M), and phosphoryl (S, T, Y) as variable modifications; default peptide and fragment tolerance (maximum normal distribution of 10 ppm and 20 ppm, respectively); and false discovery rate ≤ 4%. The average MS signal responses of the three most intense peptides protein (Hi3) were used for the absolute quantitation according with the method described by Silva et al. [31]. Results generated from the Progenesis software were exported to ∗.csv files in order to the next analysis.

Identified peptides by mass spectrometry were analysed for the recognized PTM. Next, we selected all serine phosphorylated peptides from each group and proteins corresponding to these phosphorylated peptides were recognized. Besides, we use the Uniprot and Phopsphosite Plus software (http://phosphosite.org) to confirm whether phosphorylation sites match against sites detected by MS and posteriorly, we search the cellular function of each protein. Subsequently, we compared the serine phosphorylated proteins of all the groups to detect phosphoproteins that coincided or not among the groups, mainly those phosphoproteins identified in LSIL, HSIL, and CC but that are not phosphorylated in the control group.

## 3. Results

### 3.1. Serine Phosphoproteins in Biopsies from Patients with Cervical Intraepithelial Lesions

**Figure 1:**
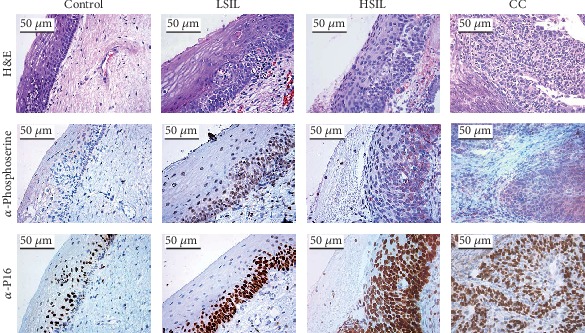
Immunohistochemical analysis of antiphosphoserine in cervical cancer and precancerous lesion tissues (×50). Positive staining of endothelial cells in healthy biopsy specimen, LSIL biopsy specimen, HSIL biopsy specimen, and CC biopsy specimen. Antiphosphoserine immunostaining. P16 immunostaining.

To corroborate the different grades of cervical lesions, we performed the histological evaluation by hematoxylin and eosin (H&E) stain. The control group showed the normal epithelial stratification (Figure 1), whereas in LSIL samples, we observe atypical morphological changes in the cell and an epithelial thickening, and they also presented cytopathic alterations in lower layers; however, mature and differentiated cells can be observed (Figure 1). HSIL specimens exhibited a larger number of abnormal mitotic figures and a greater loss of cell stratification; in addition, few cell differentiation and marked nuclear abnormalities were observed (Figure 1). Tissue with CC showed loss of cell polarity and stratification, nuclei with increased size, and koilocytes that diffuse to the muscle tissue (Figure 1).

When we performed immunohistochemistry using an antiphosphoserine antibody, we observed positive cells in all groups, but with considerable differences; in healthy tissues, a basal expression was detected, and the staining was mainly in the basal layer (Figure 1). Interestingly, the number of positive cells increased in correlation with the grade of cervical lesion, where in CC tissues, the immunoreaction was more evident when loss of cell stratification occurred (Figure 1). In addition, we analysed immunostaining with anti-P16, which is a biomarker for the detection of CC with HPV infection [32]. Results confirmed the presence of HPV in the analysed samples (except in healthy samples), as well as an increase in the number of positive cells that correlated with the different degrees of cervical lesions (Figure 1). Moreover, we confirmed the presence of HPV in those samples by PCR using MY09/11 primers [33] (data not shown). Thus, probably the phosphorylation of serine residues is probably promoted by infection with HPV [34].

### 3.2. Identification of Phosphorylated Proteins from Patients with Different Injury Grade

**Figure 2:**
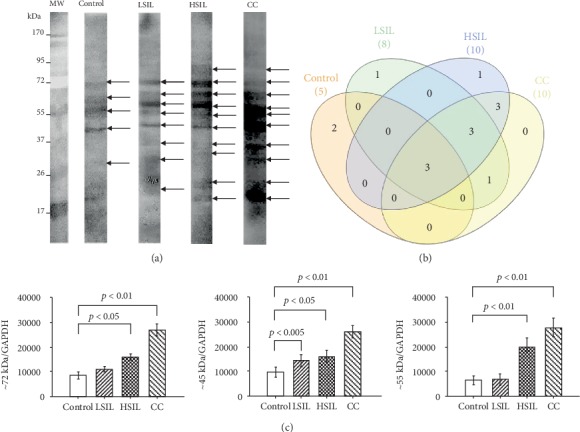
Expression of phosphoserine in different groups by western blotting. (a) Sample lysates were subjected to SDS-PAGE followed by western analysis with antiphosphoserine in the control (healthy), LSIL, HSIL, and CC samples. (b) Total number of bands detected that are present in all groups. (c) Densitometric analysis of ∽72, ∽55, and ∽45 kDa bands in all groups, the relative expression in cervical lesions were determined by Student's *t*-test, considering *p* values <0.05 as statistically significant.

To determine the profile of serine phosphorylated proteins in the different cervical lesions, protein extracts of samples obtained with cytobrushes were evaluated by western blot using the antiphosphoserine antibody. Eight bands with molecular weights from ~21 to ~72 kDa were detected in the healthy group, whereas in LSIL, HSIL, and CC, we observed ten bands, but the recognition of most of these serine phosphoproteins was stronger in CC (Figure 2(a)). Comparing the bands detected in the different samples, three of them were found in all groups; other three bands were shared in LSIL, HSIL, and CC groups, but absent in the healthy tissues; three common bands were detected in HSIL and CC; and one band was shared between LSIL and CC group (Figure 2(b)). On the other hand, two bands in the healthy tissue and one in each precancerous lesion were exclusives of these samples (Figure 2(b)). This comparative analysis among groups contribute to the knowledge of the distribution of the serine phosphoproteins in each sample; however, the bands shared between HSIL and CC may be of great interest, since they could have an important role in the progression of cervical cancer.

As mentioned before, there are some bands shared by all samples (~72, ~55, and ~45 kDa); however, they apparently increased their serine phosphorylation in agreement with the injury grade. To confirm this assumption, we analysed their relative expression by densitometry, using GAPDH as a loading normalizer and taken the data from the healthy tissues as the arbitrary unit. Statistics confirmed that these bands significantly augmented in HSIL and CC compared with healthy samples (Figure 2(c)).

**Figure 3:**
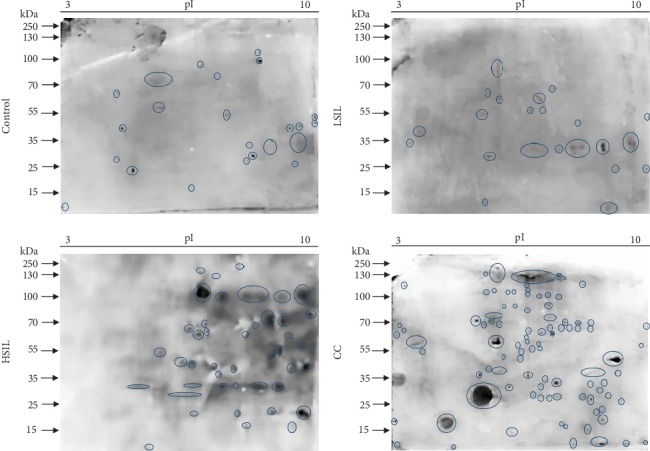
Expression of phosphoserine by combination 2DE gels and western blotting. Immunodetection of phosphoserine spots in the control, LSIL, HSIL, and CC groups.

Then, to evaluate with more detail the profile of serine phosphorylation, we performed a comparative analysis by 2D electrophoresis and western blot. Results showed a significant increase in the number of detected spots in HSIL and CC compared with the control group and LSIL (Figure 3). The antiphosphoserine antibody detected 22 spots in the control group, 23 in LSIL, 43 in HSIL, and 94 CC group (Figure 3). In addition, some spots showed a very strong recognition in HSIL and CC (Figure 3).

Thus, results obtained by immunohistochemistry and immunoblotting in 1D and 2D showed an increase in protein phosphorylation in serine residues in precancerous lesions and a greater phosphorylation when these lesions progress to CC.

### 3.3. Identification of Phosphorylated Proteins by Mass Spectrometry

The above results showed similar number of serine phosphoproteins between control and LSIL and a considerable increase in CC. Thus, we decided to identify the serine phosphorylated peptides in the control and CC tissues by LC-ESI-HDMS^E^ analysis. In the healthy group, 2767 peptides corresponding to 214 proteins were identified, and from a thorough and manual analysis of the peptides, we detected 84 phosphorylated peptides, of which 42 were phosphorylated in serine residues representing to 30 phosphoproteins. In CC, we detected 14,804 peptides from 989 proteins; we also found 987 phosphopeptides, of which 507 were phosphorylated in serine residues and related to 289 phosphoproteins. In summary, LC-ESI-HDMS^E^ data showed an increase in the expressed proteins in CC and confirmed a significant augment in the number of serine phosphorylated proteins in this cancer (Table 1).

**Figure 4:**
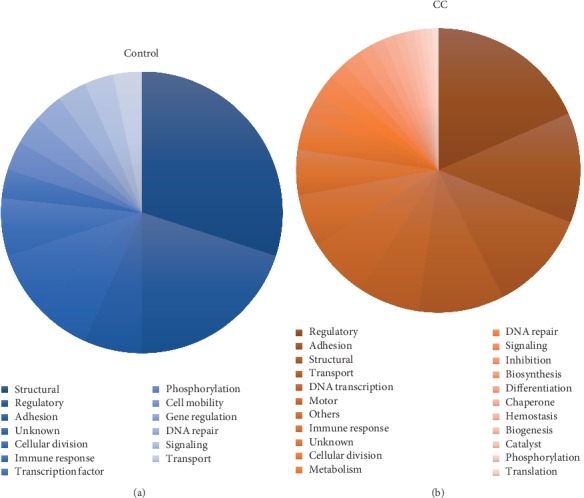
Functional annotation of serine phosphorylated proteins identified in different groups. (a) Cellular roles of serine phosphoproteins in the control group. (b) Cellular roles of serine phosphoproteins in the CC group.

Next, we investigated the cellular roles of the serine phosphorylated proteins of each group. The serine phosphoproteins detected in the control group participate in 13 different cellular functions, where the main ones were of structural (30%) and regulatory (20%) (Figure 4(a)). In the CC group, the serine phosphoproteins take part in 22 different cellular functions, mainly in regulatory (18%), adhesion (13%), of structural (12%), and of transport (10%) (Figure 4(c)), and finally, the serine phosphoproteins of both groups were compared, and we found 45 proteins (Supplementary [Supplementary-material supplementary-material-1]), including HSPB1 (also known HSP27), Clusterin, and citokeratins 8 and 19 (KRT8, KRT19), that only contain serine phosphorylated residues in the CC samples (see Supplementary [Supplementary-material supplementary-material-1]).

## 4. Discussion

Here, we show a possible correlation between the increase in phosphorylation of protein in serine residues and the injury degree of cervical lesions in samples obtained with cytobrushes. Unlike the obtention of biopsies, which is an invasive and long-time-consuming procedure, the use of cytobrush is a noninvasive technique that can be coupled to the Pap Test. In these samples, we observed that the number of serine-phosphorylated proteins increased depending to the lesion grade. We also noticed an augment in the phosphorylation of the shared bands detected by the antibody against phosphoserine (72, 55, and 45 kDa) whose densitometry was highest in tissues that evolve to cancer. Moreover, immunoblotting in 2D electrophoresis corroborated the augment of serine-phosphorylated spots, mainly in HSIL and CC. With these data, we decided to identify by mass spectrometry the serine-phosphorylated proteins in the control and CC groups. We obtained 30 and 289 serine phosphorylated proteins in the healthy and CC group, respectively. Interestingly, in the comparison of serine phosphorylated proteins between control and CC group, we found 45 proteins expressed in both groups, but only serine phosphorylated in the cancerous lesion, of which we highlight HSPB1 (also known HSP27), Clusterin, KRT8, and KRT19.

Heat-shock protein 27 (HSP27 or HSPB1) plays an important role in several types of cancer, because it acts as an antioxidant and as an apoptosis inhibitor, protecting cells from cell death [35]. Overexpression of HSP27 has been associated with poor prognosis in gastric, liver, prostate, breast, osteosarcoma, and cervical cancer [35, 36]. Various stimuli lead to the phosphorylation of HSP27 at serine residues at position 15, 78, and 82, and phosphorylated HSP27 (p-HSP27) is often associated with changes on its oligomerization and biological functions [37]. Accumulated evidence suggested that HSP27 plays an important role in radiotherapy- and chemotherapy-induced apoptosis [38]. Santiago-O'Farrill et al. [39] evaluated p-HSP27 and its correlation with autophagy-induced chemotherapy on osteosarcoma cells lines; according with their results, authors suggested that p-HSP27 could be a beneficial predictive biomarker for combination therapies for osteosarcoma patients. Other studies on pancreatic cancer mention that a higher expression of p-HSP27 correlates with a better survival to treatment with chemotherapy; therefore, it has been proposed to evaluate the expression of p-HSP27 to predict the prognosis of this cancer [40–42]. However, other authors reported that the high expression of p-HSP27 helps in the resistance to the treatment with gemcitabine; thus, they propose to use p-HSP27 as a biomarker to predict the response of patients with pancreatic cancer to treatment with this drug [43]. Studies in cervical cancer have only evaluated changes in the expression of nonphosphorylated HSP27; nevertheless, in HeLa cells, it has been reported that phosphorylated HSP27 is involved in the apoptosis induced by TNF-*α* mediating its interaction with TAK1 and regulating the posttranslational modifications of TAK1 [44]. In another study [45], it was observed that suppression of p-HSP27 potentiated the TRAIL-induced apoptosis and attenuated TRAIL-triggered activation of Akt and ERK survival pathways by suppressing the phosphorylation of Src. In addition, a physical binding between *β*-arrestin2 and Src was detected; thus, authors speculated that *β*-arrestin2 could recruit the formation of complex of p-HSP27/*β*-arrestin2/Src in response to TRAIL, resulting in the activation of survival signaling. Therefore, our results accumulate evidence about a probable participation of p-HSP27 in the progression of CC.

On the other hand, the functions of Clusterin in cells are partially known, and an involvement of this protein in apoptosis through complexing with Ku70 autoantigen (nCLU, proapoptotic) [46] or interfering with Bax activation (sCLU, antiapoptotic) has been suggested [47]. In cancer, the overexpression of sCLU mediates in part the activation of the PI3K/Akt pathway and increased the per se phosphorylation of Akt, with the consequent Akt-induced phosphorylation of Bad, thus, inhibiting TNF*α*-induced apoptosis [48]; otherwise, in prostate cancer, proapoptotic nCLU decreased, while antiapoptotic sCLU increased [49, 50]. In addition, an augment in the Clusterin expression has been demonstrated in breast [51], ovarian [52], colorectal [53], and pancreatic [54] cancer and that Clusterin plays an important role in the cell survival in response to chemotherapy in these cancer types [55–57]. In Hela cells, a study from Kim et al. [58] showed that the upregulation of LXR O-GlcNAcylation enhances the sCLU expression through an increased expression of SREBP-1, which induces drug resistance in cervical cancer cells. On the other hand, Lee et al. [59] examined the proapoptotic effect of PACAP in cervical cancer cells and propose that PACAP interferes with CLU-mediated cancer cell survival. Nonetheless, so far, studies have not been done on Clusterin phosphorylation and the role it plays on CC, so future studies on Clusterin phosphorylation would be of great importance to determine the roles of this phosphoprotein in cervical tumorigenesis.

Phosphorylation is considered a major regulator of keratins; this posttranslational modification modulates their reorganization under stress and intrinsic properties, such as solubility, other PTM, and structural conformations [60]. Keratin 8 (KRT8) has been studied as biomarker in cancer [61]; this cytokeratin is phosphorylated in serine 23, 73, and 431 [62]. Phosphorylated KRT8 in serine 431 plays an important role in human pancreatic and gastric cancer cells, because it induces keratin reorganization and consequently enhanced migration of tumor cells [63]. Arentz et al. [64] observed in colon cancer that phosphorylated KRT8 promotes tumor cell survival and progression. On the other hand, Tiwari et al. [65] analysed the importance of the phosphorylation of KRT8 in serine residues 73 and 431 in skin squamous cell carcinomas (skin-SCC). The analysis showed that a significant proportion of total phosphoproteome is associated with the migratory, proliferative, and invasive potential of these cells. However, the participation of phosphorylated KRT8 in progression in CC is unknown, so it is necessary to perform future research to determine its role.

Keratin 19 (KRT19) pairs with KRT7 in simple epithelia and with KRT8/18 in stratified squamous epithelial cells [66]. A previous study showed high expression levels of KRT7 and KRT19 in Cervical Intraepitelial Neoplasm grade 3 (CIN3) and squamous cell carcinoma (SCC), supporting the idea that KRT19 may promote E7 oncoprotein production, contributing carcinogenic events [67]. The phosphorylation of KRT19 has not been fully studied, and the phosphorylation of KRT19 in serine residues 35 plays a role in keratin filament assembly [68]; besides, the phosphorylation of KRT19 in the tyrosine residue 391 has been reported [69]. Although the importance of the possible phosphorylation of KRT19 in serine residues has not been sought, the phosphorylation in serine residue 14 has found hyperphosphorylated in breast, colon, ovarian cancer, lung adenocarcinoma, and uterine corpus endometrial carcinoma (UCEC) [70]. Our results identified the phosphorylation of KTR19 in serine residues; despite this, more extensive research is needed to determine the probable role that phosphorylated KRT19 plays in the progression of cervical cancer. In addition, the serine phosphorylated KRT8 and KRT19 could interact between them and to participate on the deregulation of several cellular functions leading to cellular growth.

In addition to serine phosphorylation of KRT8, KRT19, Clusterin, and HSP27, it has been suggested that phosphorylation in serine of other proteins identified in this work, such as Apolipoprotein B100, Serpin B3, Cofilin 1, and Lactotransferrin, is involved in different biological processes such as signaling, cell migration, and apoptosis. Phosphorylation of Serpin B3 was reported in cervical mucus using Pro-Q Diamond, a phosphor-specific stain [71], but it has not been determined if the phosphorylation of this protease inhibitor plays a role in cervical cancer, although due to its decreased expression, serpin B3 is considered a biomarker in cervical cancer [72]. Phosphorylation in serine residues of Cofilin 1 participates in its function regulation; phosphorylation of serine 3 inactivates this protein, and phosphoserine 24 may prevent the recognition of its nuclear localization signal [73]. This protein is upregulated in the presence of the enterovirus 71 in rhabdomyosarcoma [74], and the hyperphosphorylation of serine 7 was detected in various types of cancer [70], suggesting that Cofilin 1 has a role in cancer development. Interestingly, we found the phosphorylation of serine 7 in Cofilin 1 of CC.

On the other hand, we detected serine phosphorylation in several actin-associated proteins, such as Adenylyl cyclase-associated protein 1, Alpha-actinin-4, Alpha-enolase, Annexin A3, Beta-enolase, Carbonic anhydrase 1, Centrosome-associated protein 350, Ceruloplasmin, Dynein heavy chain 10, 9, 3_ axonemal, Fibrinogen beta chain, Gelsolin, Plastin-2, Plectin, Prolow-density lipoprotein receptor-related protein 1, Protein unc-13 homolog B, Protocadherin Fat 1, Rab11 family-interacting protein 1, and mitochondrial superoxide dismutase (Mn), that probably contribute to the dynamics of the cytoskeleton for cell migration, cell proliferation, and cell cycle.

## 5. Conclusions

Overall, in this study, we showed a correlation between the increment of phosphorylated proteins in serine residues and progression of CC. We discussed the possible participation of several serine phosphoproteins in cervical tumorigenesis. These results provide a support for future studies focused on these phosphoproteins and their possible correlation with the progression of cervical lesions in patients infected with HPV-HR.

## Figures and Tables

**Table 1:** Total number of serine phosphorylated proteins identified by LC-ESI-HDMSE analysis.

Group	Total peptides	Total proteins	Total phosphopeptides	Serine phosphopeptides	Serine phosphoproteins
Control	2767	214	84	42	30
CC	14,804	987	933	507	289

## Data Availability

All data generated of analysis during this study are included in this published article.

## Supplementary Materials

Supplementary Materials Supplementary Table 1: list of proteins on common of serine phosphoproteins of the CC group compared to nonphosphorylated proteins of the control group.
