# Supplementary material for: Protein Phosphorylation in Serine Residues Correlates with Progression from Precancerous Lesions to Cervical Cancer in Mexican Patients
Source: Biomed Res Int. 2020 Apr 2;2020:5058928. doi: 10.1155/2020/5058928 (PMC7157794; doi:10.1155/2020/5058928)
Supplement: Supplementary Materials — Supplementary Table 1: list of proteins on common of serine phosphoproteins of the CC group compared to nonphosphorylated proteins of the control group. [file 5058928.f1.pdf]

Supplementary Table 1. List of proteins on common of serine phosphoproteins of CC group compared to non-phosphorylated proteins of control group.

**NCBI-IP**	**Proteins CC (Phospho)/ Control (No phospho)**	**Peptides count**	**Coverage (%)**	**Confidence score**
P60709<br>P63261<br>A0A2R8Y793<br>I3L1U9<br>I3L3I0<br>I3L4N8<br>P63267-2<br>A0A2R8YGF8<br>E7EVS6<br>G5E9R0<br>I3L3R2<br>J3KT65<br>K7EM38<br>A0A2R8YEA7<br>B8ZZJ2<br>C9JTX5<br>C9JUM1<br>C9JZR7<br>F8WB63<br>A0A2R8YFE2<br>F8WCH0<br>Q9BYX7	Actin_ cytoplasmic 1	68	2.933	272.4554
Q01518<br>Q01518-2<br>Q5T0R1	Adenylyl cyclase-associated protein 1	15	5.89	75.7606

Q5T0R9<br>Q5T0R2<br>Q5T0R3<br>Q5T0R4<br>Q5T0R5<br>Q5T0R6<br>Q5T0R7				
P01011<br>G3V3A0<br>G3V595<br>P01011-2	Alpha-1-antichymotrypsin	31	2.364	139.9043
O43707<br>O43707-2<br>H9KV75<br>P12814<br>P12814-2<br>P12814-3<br>P12814-4<br>F5GXS2<br>O43707-3<br>H7C144<br>G3V2W4<br>H0YJ11<br>H0YJW3<br>H7C5W8<br>K7EJH8<br>G3V2N5<br>A0A087WSZ2<br>G3V2E8<br>G3V2X9<br>G3V5M4	Alpha-actinin-4	36	8.56	250.2948

Q08043				
P06733<br>A0A2R8Y6G6<br>P06733-2<br>K7EM90<br>A0A2R8Y798<br>A0A2R8Y879<br>A0A2R8YEG5<br>A0A2R8YEM5<br>K7ERS8<br>P13929-3	Alpha-enolase	30	6.91	189.5342
P12429<br>D6RA82<br>D6RFG5<br>D6RCA8<br>D6RAZ8	Annexin A3	27	3.096	126.0585
P04114	Apolipoprotein B-100	61	3.15	256.5224
P13929<br>E5RG95<br>E5RGZ4<br>E5RI09<br>K7EKN2<br>K7EPM1<br>P13929-2	Beta-enolase	10	6.91	61.902
P00915<br>E5RFE7<br>E5RHP7<br>E5RJI8<br>E5RG81	Carbonic anhydrase 1	36	9.19	138.346

E5RH81<br>E5RJF6<br>H0YBE2				
P08311	Cathepsin G	25	8.23	73.2588
Q5VT06<br>H0Y7F7<br>H0Y6Q4<br>A2A4F6<br>E9PIK0<br>H0YD38	Centrosome-associated protein 350	15	1.155	54.7112
P00450<br>E9PFZ2<br>H7C5R1<br>D6RE86	Ceruloplasmin	16	1.50	81.5754
P10909<br>P10909-2<br>P10909-4<br>P10909-5<br>P10909-3<br>H0YAS8<br>H0YC35<br>E7ERK6<br>H0YLK8<br>E5RG36<br>E5RGB0<br>E5RH61<br>E5RJZ5<br>E7ETB4	Clusterin	7	5.12	31.2289
P23528<br>E9PK25<br>E9PP50	Cofilin-1	11	7.23	44.157

G3V1A4<br>E9PLJ3<br>E9PQB7<br>E9PS23<br>Q9Y281<br>Q9Y281-3				
P12111<br>E7ENL6<br>P12111-2<br>P12111-3<br>P12111-4<br>P12111-5<br>C9JNG9	Collagen alpha-3(VI) chain	14	1.10	49.3082
A8TX70<br>A8TX70-2<br>E9PAL5<br>H0Y935	Collagen alpha-5(VI) chain	18	0.42	76.5644
Q8IVF4<br>A0A1C7CYW8<br>A0A0J9YY17	Dynein heavy chain 10_ axonemal	21	0.25	86.0591
Q8TD57<br>Q8TD57-3<br>Q8TD57-2	Dynein heavy chain 3_ axonemal	16	0.85	52.6606
Q9NYC9	Dynein heavy chain 9_ axonemal	40	0.95	147.4929
P02675<br>D6REL8	Fibrinogen beta chain	44	3.66	257.9515
P06396<br>A0A0A0MS51<br>A0A0A0MT01<br>P06396-2	Gelsolin	13	5.498	84.8484

P06396-3<br>P06396-4				
P04792<br>F8WE04<br>C9J3N8	Heat shock protein beta-1	29	4.87	137.4486
P0DOY2<br>P0DOY3<br>A0M8Q6<br>P0CF74<br>A0A0B4J231<br>B9A064<br>P0CG04	Immunoglobulin lambda constant 2	18	16.98	63.314
P13645	Keratin_ type I cytoskeletal 10	60	7.36	333.4504
P02533<br>K7ENW6<br>Q7Z3Y9	Keratin_ type I cytoskeletal 14	63	11.86	295.7665
P19012<br>A8MT21<br>P19012-2<br>C9JTG5<br>Q04695<br>F5GWP8<br>K7EPJ9	Keratin_ type I cytoskeletal 15	98	6.789	415.8429
P08779	Keratin_ type I cytoskeletal 16	47	13.74	377.219
P08727<br>C9JM50<br>K7EMS3	Keratin_ type I cytoskeletal 19	73	2	450.5063
P04264<br>A0A1W2PQU7<br>A0A1W2PRT3<br>A0A1W2PS58	Keratin_ type II cytoskeletal 1	110	6.36	412.4752

K7EPI4<br>Q6KB66<br>Q6KB66-2<br>Q6KB66-3				
P19013<br>H0YIC5	Keratin_ type II cytoskeletal 4	134	2.43	518.8999
P02538	Keratin_ type II cytoskeletal 6A	90	6.737	708.9958
P48668<br>Q01546<br>Q9NSB2<br>A0A087X106<br>B4DIR1<br>F8VS61<br>H0YHD9<br>K7EKH9<br>K7ELP4<br>O43790<br>P78385<br>Q14533<br>Q14CN4<br>Q14CN4-2<br>Q14CN4-3<br>U3KPR1	Keratin_ type II cytoskeletal 6C	150	4.96	700.0887
O95678	Keratin_ type II cytoskeletal 75	52	2.54	293.3287
Q5XKE5<br>H0YID6	Keratin_ type II cytoskeletal 79	38	6.542	172.8744
P05787<br>P05787-2<br>F8VUG2<br>F8W1U3<br>A0A1W2PR46	Keratin_ type II cytoskeletal 8	44	7.45	274.6775

A0A1X7SBR3<br>A0A1X7SCE1<br>K7EJU1<br>K7EMP8<br>P14136<br>P14136-2<br>P14136-3<br>F8VP67				
P02788<br>E7EQB2<br>E7ER44<br>P02788-2<br>C9JCF5	Lactotransferrin	75	3.098	290.4927
P30740<br>P30740-2	Leukocyte elastase inhibitor	37	3.43	163.1504
P13796<br>P13796-2<br>Q5TBN3<br>A0A0A0MSQ0<br>P13797<br>P13797-2<br>P13797-3<br>U3KQI3	Plastin-2	44	8.77	247.5619
Q15149;Q15149-<br>2;Q15149-<br>3;Q15149-<br>4;Q15149-<br>5;Q15149-<br>6;Q15149-<br>7;Q15149-	Plectin	40	0.469	167.2703

8;Q15149-<br>9;E9PMV1<br>H0YDN1				
Q07954	Prolow-density lipoprotein receptor-related protein 1	15	1.25	66.4491
A0A1B0GUS7	Protein unc-13 homolog B	8	0.967	30.6686
A0A087WVP1<br>Q14517	Protocadherin Fat 1	9	0.545	27.2938
Q6WKZ4<br>Q6WKZ4-1	Rab11 family-interacting protein 1	2	1.247	7.1718
P29508<br>P29508-2<br>H0Y5H9<br>C9JZ65	Serpin B3	39	9.487	310.5015
P04179<br>P04179-2<br>P04179-4<br>P04179-3<br>F5GYZ5<br>F5H3C5<br>F5H4R2<br>G8JLJ2	Superoxide dismutase [Mn]_ mitochondrial	6	15.765	42.06
